# An uncommon presentation of an erupted compound odontoma in the anterior maxilla: A case report

**DOI:** 10.1016/j.radcr.2025.04.098

**Published:** 2025-05-24

**Authors:** Narges Mirzania, Mehdi Hosseinzadeh, Elaheh Rahimipour, Mahsa Mohammadpour

**Affiliations:** Department of Oral and Maxillofacial Radiology, School of Dentistry, Shahid Beheshti University of Medical Sciences, Tehran, Iran

**Keywords:** Odontoma, Odontogenic tumors, Cone-beam computed tomography, Oral surgical procedures

## Abstract

Odontomas are the most common benign odontogenic tumors, classified into compound (tooth-like structures) and complex (amorphous dental tissue) subtypes. While typically asymptomatic, erupting odontomas are rare and can cause diagnostic and management challenges. This case involves a 15-year-old male with an erupted compound odontoma in the maxillary anterior region, presenting primarily with aesthetic concerns. Clinical and CBCT evaluations confirmed the diagnosis, and surgical excision followed by histopathological analysis revealed the characteristic features of a compound odontoma. The rarity of erupting odontomas underscores the importance of thorough diagnostic evaluations, including CBCT imaging, which aids in distinguishing them from other odontogenic lesions. A multidisciplinary approach involving oral surgery and orthodontics is essential for optimal functional and aesthetic outcomes. Although the exact etiology remains unclear, early diagnosis and intervention are crucial to preventing complications. This case highlights the need for increased awareness and further research into the mechanisms underlying this rare phenomenon.

## Introduction

Odontomas are the most common benign odontogenic tumors of epithelial and mesenchymal origin[1]. These lesions contain tooth-like structures, including enamel, dentin, and, in some cases, cementum and pulp tissue [2]. The term odontoma refers to lesions composed of all dental tissues, which are classified into 2 types: complex and compound odontomas [3]. Complex odontomas consist of all dental tissues arranged in a disorganized pattern. Compound odontomas contain dental tissues arranged in an orderly pattern, resembling miniature teeth [4].

Odontomas are generally considered hamartomas, though their exact etiology remains unclear [5]. Compound odontomas can develop anywhere in the dental arches but are most commonly found in the anterior maxilla [2]. Due to their slow, asymptomatic growth, they are often detected incidentally during routine radiographic examinations, typically in the second or third decade of life[6].

Radiographically, odontomas appear as radiopaque masses with multiple small calcified structures resembling miniature teeth, surrounded by a radiolucent rim [2,7]. Although odontomas are well-documented in the literature, *erupting* odontomas are rare. In this report, we present a case of an erupted compound odontoma in a young male, highlighting its clinical and radiographic features.

## Case report

A 15-year-old male presented with a tumor-like structure in the upper front teeth region, primarily causing an aesthetic concern. The patient reported no pain or history of infections. His medical history was unremarkable.

**Fig. 1:**
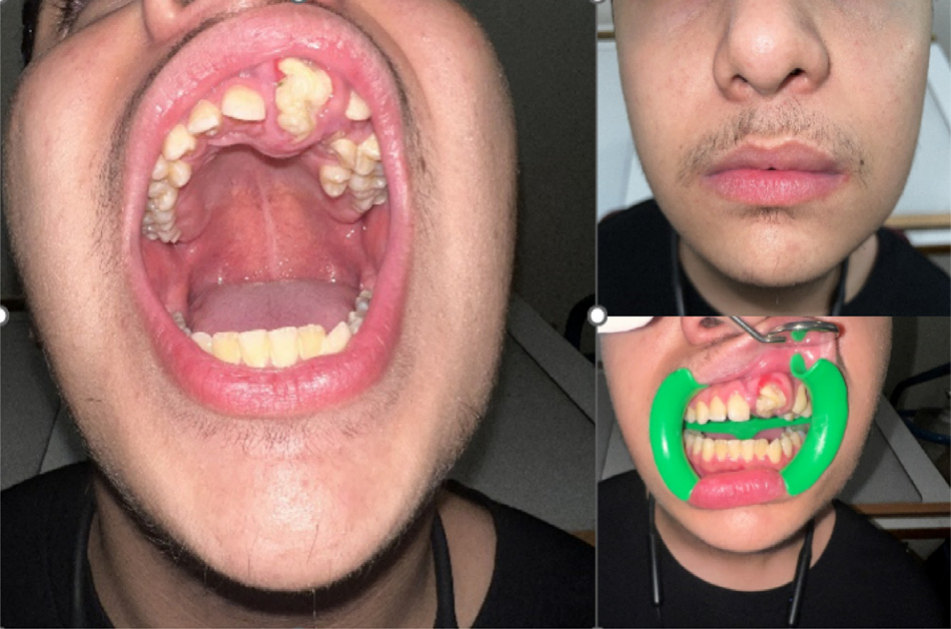
Large, whitish, malformed tooth-like structure in the maxillary anterior region, with eruption to occlusion.

**Fig. 2:**
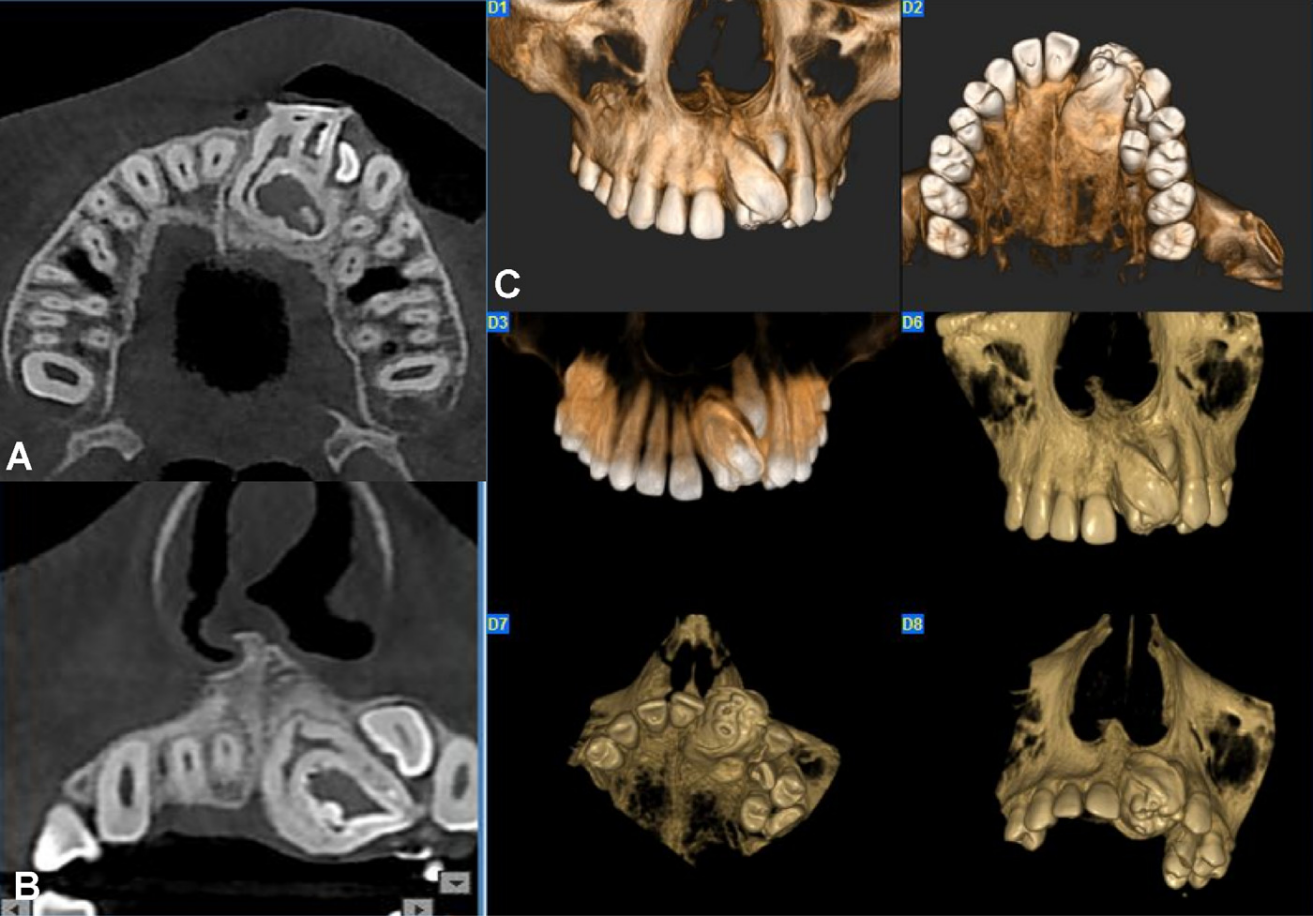
Cone beam computed tomography images showing a large compound odontoma with thin radiolucent rim surrounding the mineralized tissue, and interference with the eruption of associated teeth.: (A) axial view, (B) coronal view, and (C) three-dimensional reconstruction of tumor.

Intraoral examination revealed a large, whitish, tooth-like structure in the anterior maxillary region, accompanied by expansion of the cortical plate and displacement of adjacent teeth (Fig. 1). Cone-beam computed tomography (CBCT) demonstrated a well-defined mixed lesion, with radiodensity similar to that of dental tissues, located coronal to the crowns of the impacted left maxillary central and lateral incisors. These findings were consistent with an erupted compound odontoma. The patient underwent surgical removal of the lesion. The left central incisor was preserved and realigned through orthodontic treatment (Fig. 2).

## Discussion

Odontomas are the most common benign odontogenic tumors. Numerous case series have reported that most odontomas are diagnosed within the first 2 decades of life, with a higher prevalence in males than females, at a ratio of approximately 1.5:1 to 1.6:1 [8]. The compound type is more frequently found in the anterior maxilla, accounting for 62% of cases [9], which aligns with our case.

Odontomas are often associated with dental abnormalities, including impaction, malposition, diastema, aplasia, malformation, and devitalization of adjacent teeth. However, the eruption of an odontoma into the oral cavity is rare [9]. In our case, a compound odontoma was found to have erupted in the anterior maxilla.

Odontomas are classified into 2 types based on their gross and radiographic features: compound and complex. Complex odontomas are less common, with an estimated ratio of 1:2 compared to compound odontomas [8]. Compound odontomas typically develop between teeth as calcified structures that mimic the organization of a normal tooth. These structures vary in size and shape and are surrounded by a narrow radiolucent zone [2]. In contrast, complex odontomas appear as disorganized masses of odontogenic hard tissue—comprising dentin, enamel, and cementum—without any resemblance to a tooth. Like compound odontomas, they are also encased by a narrow radiolucent rim [8].

Histologically, compound odontomas are relatively easy to diagnose, as they replicate the structural organization of a normal tooth within a fibrous matrix. However, mature enamel may be lost during the decalcification process and may not be visible on conventional hematoxylin and eosin-stained slides. Nonetheless, varying amounts of enamel matrix are often present, and pulp tissue may be observed in the coronal and root portions of the tooth-like structures [8].

Differential diagnosis includes:

Erupting sequestrum, which is an uncommon condition usually associated with the permanent mandibular first molar. It appears as a small, irregular bone spicule, and histopathological examination reveals nonvital bone [10]. Peripheral osteomas are benign bone tumors that can occur in the maxillofacial region. While they are radiopaque and well-circumscribed, they lack the tooth-like structures and organized dental tissue characteristic of compound odontomas. They are described as slow-growing and asymptomatic, with dense radiopacity but without odontogenic origin or associated impacted teeth [10]. Supernumerary teeth most commonly involve the premaxilla and are typically conical in shape [11]. Ameloblastic fibro-odontomas can resemble compound odontomas both radiographically and clinically, presenting as mixed radiolucent-radiopaque lesions associated with unerupted teeth. However, ameloblastic fibro-odontomas are typically more aggressive, often presenting with pain, delayed eruption of teeth, and bone expansion [9]. The incidence of ameloblastic fibro-odontomas is much lower than odontomas.

## Conclusion

While odontomas are generally asymptomatic and rarely erupt into the oral cavity, this case of an erupted compound odontoma in a young male patient in the anterior maxilla adds to the limited reports of such occurrences. It is essential to differentiate odontomas from other odontogenic tumors, such as ameloblastic fibro-odontomas, to ensure appropriate diagnosis and management.

## Patient consent

Written, informed consent for publication of this case report was obtained from the patient. The patient has reviewed the case details and has agreed to its publication.
